# Editorial: Experimental Models of Epilepsy and Related Comorbidities

**DOI:** 10.3389/fphar.2019.00179

**Published:** 2019-03-04

**Authors:** Ayanabha Chakraborti, Mohd. Farooq Shaikh, Annamaria Vezzani, Jafri Malin Abdullah

**Affiliations:** ^1^Department of Surgery, School of Medicine, University of Alabama at Birmingham, Birmingham, AL, United States; ^2^Neuropharmacology Research Laboratory, Jeffrey Cheah School of Medicine & Health Sciences, Monash University Malaysia, Bandar Sunway, Malaysia; ^3^Department of Neuroscience, Istituto di Ricerche Farmacologiche Mario Negri IRCCS, Milan, Italy; ^4^Brain Behaviour Cluster & Department of Neurosciences, School of Medical Sciences, Hospital Universiti Sains Malaysia, Universiti Sains Malaysia, Kota Bharu, Malaysia

**Keywords:** epilepsy, comorbidities, animal models, anticonvulsants, inflammation in epilepsy

Epilepsy is a chronic neurological disease and patients with epilepsy have an increased risk for cognitive, behavioral, and psychosocial disorders that can adversely impact the quality of life. Various factors like common underlying predisposition, mechanisms underlying epileptogenesis, direct effects of seizures, and adverse effects of anti-seizures drugs or other non-pharmacological therapies may play a causal role in such epilepsy associated comorbidities. New and validated screening approaches as well as novel therapeutic approaches are warranted, that may help with the early detection and treatment of comorbid conditions. Experimental models of epilepsies can play a critical role for improving our understanding of not only of the fundamental mechanism of epileptogenesis but also to generate new insights regarding the relationship between epilepsy and related comorbidities.

This Research Topic compiles 12 articles, including 3 reviews, 1 mini-review, and 8 original research contributions from leading scientists in the field. The collection of papers on this topic offers an up-to-date overview of current knowledge and approaches for studying epilepsy and related comorbidities. The articles highlight the pivotal role of numerous experimental and clinical studies that have elucidated novel mechanistic insights about the pathophysiology of these conditions. Translational perspective of research in this domain as well as important methodological issues and some emerging hypotheses in the field are also highlighted. Recent progress in the pharmacotherapy and management of these conditions have also been critically analyzed and discussed. The content of each of these articles is summarized below.

The systematic review on non-mammalian models of epilepsy research by Arief et al., describes the role of non-mammalian models (i.e., Zebrafish, Drosophila, *C. elegans* etc.) for better understanding the mechanisms of epileptogenesis as well as for screening compounds with potential anti-seizure properties. This review highlights that a more comprehensive evaluation of these currently under-utilized non-mammalian models may help preclinical epilepsy research progress.

The article by Muke et al., reports on the anti-convulsive potential of nasally administered coumarin fraction of medicinal plant *Eclipta alba* using PTZ induced kindling model. Their mechanistic studies showed that the efficacy of coumarin nasal formulation (CNF) was due to its ability to reduce oxidative damage and neuroinflammation. These exciting findings suggest the promising role of CNF as a therapeutic modality for epilepsy and related comorbidities.

Sleep disorders and epilepsy share a complex reciprocal relationship. The review article by Kumar et al., focuses on the current status of our understanding of the pathogenesis and therapeutic avenues related to three of the most common type of epilepsy that occur during sleep, namely hypermotor epilepsy (SHE), benign partial epilepsy with centrotemporal spikes (BECTS), and Panayiotopoulous syndrome (PS). The article also discusses the recent advances in the pharmacotherapy of these conditions.

Zeng et al., in their original research article elucidate the effects of Ginsenoside compound K (GCK), the key metabolite of Ginsenoside Rb1, Rb2, and Rc, on acute seizure and the potential mechanism underlying its effects. Their study showed that that GCK exerts its effect by enhancing the expression of proteins related to GABAergic inhibition.

Currently available anti-seizure drugs (AEDs) raise several concerns related to lack of efficacy in a significant proportion of epilepsy patients and adverse events as well as their limited supply in Countries with poor resources and high costs. Over the years, traditional herbal medicines have occupied a prominent role in the treatment and management of epilepsy. However, robust experimental evidence related to the effectiveness and side effects of such herbal medications are limited. The article by Moto et al. showed that aqueous extract of *C. quadrangularis*, a plant belonging to family *Vitaceae* and traditionally well-known for its anticonvulsive properties, exerts antiepileptic as well as anxiolytic effects in the pilocarpine model of epilepsy. The authors also report that the neuroprotective effect of this herbal extract is mediated by its antioxidant properties as well as through modulation of GABAergic transmission.

The genetically epilepsy-prone rat (GEPR) represents an useful animal model for studying mechanisms of epilepsy especially for genetically determined seizure predisposition. The GEPR shows heightened sensitivity to convulsant drugs as well as to kindling, hyperbaric, and hyperthermic seizures. Aguilar et al., tested male and female genetically epilepsy-prone rat (GEPR-3), a GEPR strain that exhibits inherited susceptibility to tonic-clonic seizures, in a battery of behavioral tests and showed that GEPR-3s showed increased anxiogenesis in multiple behavioral tests of anxiety. These interesting new data underscores the importance of genetic influence that may underlie both the seizure disorder and epilepsy associated comorbidities.

Dravet syndrome (DS) is a severe form of epilepsy formerly known as severe myoclonic epilepsy of infancy (SMEI) that appears in infancy or early childhood as frequent febrile seizures. As the condition progesses, it also leads to other types of seizures like monoclonus and status epilepticus. Griffin et al., presents the state-of-the-art on DS preclinical animal models with a focus on seizure phenotypes and behavioral comorbidities. The authors have critically analyzed the use of these models for drug screening with an emphasis on assay protocols and pharmacological profiles. In particular, the article provides a detailed overview of the electrophysiological and behavioral drug screening assays in zebrafish and summarizes the data on 3,000 drugs screened to date. The authors advocate that this preclinical strategy offers a modality for effective drug screening especially for genetic epilepsy.

In their original research article, Ye et al., outline new findings about the neuroprotective effect of L-3-n-butylphthalide (NBP), a compound obtained from the seed extracts of *Apium graveolens*. Using a mouse model of chronic epilepsy, they demonstrate the effectiveness of this compound in alleviating both seizure severity and the associated behavioral and cognitive alterations. Their studies further show that NBP mediates such protective effects by increasing the transcription of neuroprotective factors like, brain-derived neurotrophic factor (BDNF), and Klotho as well as by restoring the expression of neural synaptic proteins such as postsynaptic density protein 95 and glutamic acid decarboxylase 65/67. These findings suggest that NBP is a potential therapeutic agent to control epileptic seizures and related psychological and cognitive comorbidities.

Song et al., developed a new rodent model of extended hippocampal kindling to investigate the effects of three AEDs with different mode of action on spontaneous recurrent seizures. The authors showed that phenytoin and levetiracetam mitigated the spontaneous recurrent seizures whereas lorazepam reduced the severity of motor seizures, but had little effect on duration of hippocampal discharges. Although the model needs to be further characterized, it may prove valuable for assessment of novel drugs in the future.

The mini review by Kasahara et al. provides an overview of the salient features of animal models of neonatal seizures, namely hypoxia-ischemia-induced seizures and febrile seizures. Furthermore, pharmacological strategies to address epileptogenic changes have also been addressed.

The research article by Amini et al. provides interesting insights regarding the link between neuroinflammatory changes and pathophysiology of seizures. The authors studied the effects of lipopolysaccharide (LPS) preconditioning in a rat model of electroconvulsive shock (ECS) induced seizures. Their data indicate LPS preconditioning exerts a protective effect on seizure duration, which was associated with enhanced expression of interferon regulatory factor (IRF3), and other genes associated to Toll like Receptor 4 (TLR4) signaling pathway as well as down-regulation of pro-inflammatory mediators in the hippocampus.

The studies by Meng Choo et al., explore the effects of ethanolic leaf extract of *Orthosiphon stamineus* in seizures using a zebrafish model. The authors report that treatment with the extract reduces Pentylenetetrazol (PTZ) induced seizures and ameliorates seizure-induced elevation of NF-kB, neuropeptide Y and TNF-α in the brain. Although he active constituent of *O stamineus* was not identified as yet, it is noteworthy that the anti-seizure effect of the extract was comparable to standard AED like Diazepam. This highlights that this herbal product may be a promising candidate for drug development in epilepsy.

We are very grateful to the basic research and clinical investigators who have contributed to this research topic. We hope that these diverse and thoughtful papers will be useful to researchers to inspire their work and stimulate new discussions in this exciting research area. These studies also highlight the avenues for advancing the field for improving treatment and quality of life of patients. Further characterization of neural circuits and signaling mechanisms that contribute to the pathogenesis of these conditions may lead to the development of safer and more effective therapeutic approaches to tackle epilepsy as well as the associated comorbidities.

## Author Contributions

AC took the initiative for the editorial write-up. MS, JA, and AV also contributed in writing, revising, and proofreading. All the authors approved the editorial.

### Conflict of Interest Statement

The authors declare that the research was conducted in the absence of any commercial or financial relationships that could be construed as a potential conflict of interest.

